# Seminal Homocysteine Levels in Men With Asthenozoospermia: Correlation With Sperm Parameters and Vitamin B_6_ Levels

**DOI:** 10.1155/tswj/5743309

**Published:** 2026-06-09

**Authors:** Shefa′ Aljabali, Roba Bdeir, Saleem Ali Banihani

**Affiliations:** ^1^ Department of Medical Laboratory Sciences, Faculty of Allied Medical Sciences, Jadara University, Irbid, Jordan, jadara.edu.jo; ^2^ Department of Medical Laboratory Sciences, Faculty of Applied Medical Sciences, Jordan University of Science and Technology, Irbid, Jordan, just.edu.jo; ^3^ Department of Basic Scientific Sciences, Al-Balqa Applied University, Al-Salt, Jordan, bau.edu.jo; ^4^ Department of Clinical Pharmacy, Faculty of Pharmacy, Jadara University, Irbid, Jordan, jadara.edu.jo

**Keywords:** asthenozoospermia, biomarker, homocysteine, male infertility, seminal plasma, sperm motility, sperm parameters, vitamin B_6_

## Abstract

In this study, we aim to assess the seminal plasma levels of homocysteine in men with asthenozoospermia compared with normozoospermic men and to investigate their correlations with sperm parameters and seminal plasma vitamin B_6_ levels. The levels of homocysteine and vitamin B_6_ were measured in 108 asthenozoospermic and 64 normozoospermic semen samples. Homocysteine levels in seminal plasma were determined using high‐performance liquid chromatography, whereas vitamin B_6_ levels were measured by liquid chromatography‐tandem mass spectrometry. The results showed that seminal plasma levels of homocysteine were significantly higher in the asthenozoospermic group than in the normozoospermic group (3.08 ± 0.320 vs. 1.23 ± 0.320 * μ*mol/L, *p* < 0.0001). Notably, the receiver operating characteristic (ROC) curve analysis demonstrated that the area under the curve was 0.999 (*p* < 0.0001), indicating a strong diagnostic performance of seminal plasma homocysteine in distinguishing asthenozoospermic men from normozoospermic men. In addition, the cutoff value was > 1.87 *μ*mol/L, with a specificity of 100.0% (95% CI: 94.3%–100%) and a sensitivity of 99.1% (95% CI: 94.9%–99.9%). In the normozoospermic group, there was no statistically significant association between seminal plasma levels of homocysteine and progressive motility (*p* = 0.675, *r* ^2^ = 0.00286) or total motility (*p* = 0.178, *r* ^2^ = 0.0291) of spermatozoa. However, in the asthenozoospermic group, progressive and total motility were inversely correlated with the seminal plasma levels of homocysteine (*p* = 0.0025, *r* ^2^ = 0.0827, *p* = 0.0048, *r* ^2^ = 0.0725, respectively). No significant correlation was found between homocysteine levels and sperm concentration, semen volume, age, or seminal plasma vitamin B_6_ levels in either group. These findings suggest that seminal plasma levels of homocysteine are associated with impaired sperm motility and may serve as a potential biomarker for asthenozoospermia. However, further studies are needed to confirm these findings.

## 1. Introduction

Male factor infertility affects approximately 30 million men worldwide [1] and can be caused by various factors such as genetic disorders, infections, hormonal imbalances, and lifestyle factors [2]. One common cause of male infertility is asthenozoospermia, a condition characterized by reduced sperm motility (progressive motility (PR) < 32% or total motility < 40%) [3]. Several cases of asthenozoospermia are idiopathic and remain poorly understood. The lack of a clear etiology has made it difficult to treat men with asthenozoospermia. Therefore, there has been considerable interest in conducting comprehensive research to pinpoint and understand the underlying factors in idiopathic asthenozoospermia [4–8], as well as in identifying reliable biomarkers for its diagnosis and management [9]. An intriguing factor highlighted in some of these studies is homocysteine [10], which has drawn considerable attention for its potential harmful impact on male reproductive function and its toxicity to sperm cells [11–13].

Homocysteine, 2‐amino‐4‐mercaptobutyric acid (C_4_H_9_NO_2_S), is a sulfur‐containing amino acid similar to cysteine but distinguished by an extra methylene group [14]. Homocysteine is an essential intermediate compound formed during methionine metabolism via the action of methyl transferases donating methyl groups (‐CH_3_) to acceptor compounds [15]. Interestingly, the metabolism of homocysteine is at a crossing point of two metabolic pathways: transsulfuration to cysteine, which requires vitamin B_6_, and remethylation to methionine, which requires vitamins B_9_ and B_12_ [15]. When homocysteine synthesis is elevated or its metabolism is compromised, it accumulates within the cell and is transferred to the extracellular fluids [15, 16]. The accumulation of homocysteine imposes a considerable risk of cellular damage and harmful systemic effects due to its toxic nature [17]. The high chemical reactivity and toxicity of homocysteine are mainly attributed to its chemical structure, which contains a carboxyl group at one end and a thiol group at the other end [17]. The sulfur atom in the thiol group is nucleophilic and can attack other electrophilic molecules [17].

Homocysteine causes cellular dysfunction through a variety of potential mechanisms, including induction of oxidative stress through the generation of free radicals and reactive oxygen species (ROS) via auto‐oxidation and nitrosylation [18], as well as interference with the synthesis of antioxidant enzymes (e.g., glutathione peroxidase) [19], altered nitric oxide bioavailability [20], inflammatory cytokine generation [21, 22], and activation of apoptosis [23].

Increasing evidence shows that homocysteine is implicated in human reproductive health. Notably, a handful of studies have focused on investigating the potential impact of homocysteine on male reproductive dysfunction, including impaired sperm quality and motility. For example, it was shown that hyperhomocysteinemia enhanced the number of defective seminiferous tubules in the testes and induced aberrant tissue remodeling in the caput area of the epididymis [13]. In addition, low serum levels of testosterone due to its impaired synthesis in Leydig cells were linked to hyperhomocysteinemia [24]. However, the link between sperm motility and homocysteine levels remains unclear and somewhat contradictory. For instance, one study found that the asthenozoospermic group had higher homocysteine levels in seminal plasma (*p* < 0.01) when compared with the control group [25]. In another study, the Spearman correlation analysis revealed that as the homocysteine level in semen decreases, the sperm velocity increases [25, 26]. Moreover, idiopathic asthenozoospermic men had considerably greater sperm cell homocysteine concentrations than fertile men, whereas seminal plasma homocysteine levels were comparable between the two groups [27]. Rezk et al. [28] found out that treating sperm cells collected from normal and subfertile men with homocysteine at different time points has differential reduction on sperm motility in both groups. In contrast, some studies reported that seminal plasma homocysteine levels were not significantly different between normal and subfertile men, including men with asthenozoospermia, or significantly correlated with sperm motility [27, 29–31]. As a result, there is a lack of consensus regarding the relationship between levels of homocysteine (especially in seminal plasma) and sperm motility. Therefore, further confirmatory research is needed to establish a clear association between homocysteine levels and impaired sperm motility.

In this study, we aim to specifically evaluate the seminal plasma levels of homocysteine and their diagnostic performance in men with asthenozoospermia compared with normozoospermic men, using a substantially overlapping human cohort and previously collected seminal plasma samples from our earlier study [32]. We also examined the potential correlations between homocysteine levels, sperm parameters, and seminal plasma vitamin B_6_. This study may contribute to the development of a low‐cost, noninvasive biomarker to assess impaired sperm motility and guide targeted treatments, ultimately improving conception outcomes for affected couples.

## 2. Materials and Methods

### 2.1. Study Subjects and Sample Collection

A total of 172 male participants were randomly recruited from adult men who visited the Assisted Reproductive Technologies (ART) unit at King Abdullah University Hospital and Al‐Qudah laboratories in Irbid province, Jordan. The participants were divided into two groups: normozoospermic men (*n* = 64) and men with asthenozoospermia (*n* = 108). According to the World Health Organization (WHO) guidelines, men with asthenozoospermia were classified based on PR, with a cutoff of less than 32% [3]. This study represents a follow‐up analysis, using a substantially overlapping cohort and previously collected samples from our earlier published study [32]. All semen specimens were provided in sterile, nontoxic plastic containers by masturbation after 3–7 days of sexual abstinence. A questionnaire was used to obtain the following information: men′s age, smoking, chronic diseases, family history, and the use of medication or vitamin B supplementation. Subjects were eligible to participate in this research study if they met the following requirements:

• No use of pharmaceutical drugs or vitamin B supplements.
• No history of any chronic diseases (e.g., cancer, cardiovascular diseases [CVDs], or diabetes).
• No family history of infertility.
• No reproductive conditions such as orchidectomy, varicocele, testicular tumor, or previous gonadotoxic treatment.

### 2.2. Ethical Considerations

The approval of this research work was obtained from the Institutional Review Board committee at Jordan University of Science and Technology. All recruited men in both locations were fully informed about the study′s objectives and analysis. Besides, study subjects voluntarily provided both verbal and written informed consent for their participation and data to be used in this research.

### 2.3. Semen Analysis and Storage

After liquefaction, each semen specimen was directly analyzed for the following characteristics: viscosity, volume, concentration, motility (both progressive and total), and morphology. These semen parameters were evaluated according to the WHO 2010 guidelines [3]. The semen viscosity was determined by measuring the thread length after aspirating the semen into a sterile graduated plastic tube. Semen specimens with a thread length of more than 2 cm were considered abnormal [3]. A sterile graduated conical tube was used to estimate the semen volume for each sample. Typically, ejaculate volume varies from 1.5 to 7.6 mL [3]. Afterward, all semen samples were centrifuged at ~2500 × g for 10 min, and the supernatants (seminal plasma) were isolated to be stored at −20°C for homocysteine and vitamin B_6_ analysis.

### 2.4. Measurement of Sperm Concentration and Motility

We used a phase‐contrast microscope (at 200× magnification) and Makler cell counting chamber (Irvine Scientific, United States) to manually assess sperm concentration and motility. To avoid sample evaporation, measurements were performed within 10–15 min after sample collection. For each semen sample, a well‐mixed drop (10 *μ*L) was loaded onto the Makler chamber to evaluate both sperm concentration and motility. Sperm concentration values were expressed in 10^6^/mL, whereas the total and PR values were calculated in percentages.

The motility of spermatozoa is graded as either PR or nonprogressive motility (NP). PR refers to the active movement of spermatozoa in a large circle or linearly. In contrast, NP refers to all other patterns of motility that lack progression, such as moving in small circles, observing only a flagellar beat, or the flagellar force barely displacing the head [3].

Approximately 200 sperm cells per replicate were evaluated to achieve greater accuracy, and the scanned fields were randomly chosen to minimize the bias in the analysis. Sperm counting was performed quickly enough to prevent any potential positive errors in the results.

### 2.5. HPLC Analysis of Homocysteine

Homocysteine levels were measured using an HPLC‐fluorescence derivatization method adapted from a previously published protocol with minor modifications [33]. In brief, a total of 100 *μ*L of each seminal plasma sample was mixed with 20 *μ*L of DTT (500 mM) and incubated for 30 min at room temperature. Then, 100 *μ*L of 10% trichloroacetic acid, containing 1 mM EDTA was added to the samples to achieve deproteinization. Samples were centrifuged at 10,000 rpm for 10 min to remove the precipitated proteins. In the next step, 100 *μ*L of each sample supernatant was mixed with 20 *μ*L of NaOH (1.55 M), 20 *μ*L of SBD‐F (1 mg/mL in borate buffer), and 240 *μ*L of borate buffer solution (0.125 M, pH = 9.5), containing 4 mM EDTA. Samples were incubated in the dark at 60°C for 60 min. After derivatization, the samples were acidified with 20 *μ*L HCl (6 N) and stored at 4°C until HPLC injection.

The analysis was conducted on a Shimadzu LC system consisting of a LC‐20AT quaternary pump, an RF‐10AXL fluorescence detector, a SIL‐20A sample auto injector, and a CTO‐20 AC oven. LC Solution software (ver. 1.25 SP3 2011 Shimadzu, Japan) was used for data acquisition and signals processing. Chromatographic separation was carried out on a LiChrospher RP‐18 × 250 mm (Merck) column with 5 *μ*m of particle size. The HPLC analysis was performed isocratically in reverse phase with monopotassium phosphate (20 mM, pH = 2.1) and 5% acetonitrile. The HPLC conditions are as follows: the flow rate was 1 mL/min, the column temperature was 30°C, the injection volume was 50 *μ*L, and the total analysis time was 20 min. The fluorescence detector was set at excitation/emission of 385/515 nm.

### 2.6. HPLC‐MS/MS Analysis of Vitamin B_6_

For protein precipitation, a 250‐*μ*L aliquot of each seminal plasma sample was mixed with chloroform (3 mL) and ethanol (250 *μ*L). Next, samples were vortexed thoroughly for 30 s before being centrifuged at 2500 g for 10 min. After centrifugation, supernatants were isolated and then transferred into Eppendorf tubes and placed in a nitrogen evaporator. After complete evaporation, pellets were resuspended in 750 *μ*L of methanol and then transferred into autosampler vials for HPLC‐MS/MS analysis.

The seminal plasma vitamin B_6_ analysis was performed on 30AD SHIMADZU Binary HPLC system coupled with an 8030 ESI‐MS/MS system (SHIMADZU Corp.) [34]. Briefly, the chromatographic separation was primarily conducted on a revered phase HPLC column—Zorbax Eclipse Plus C18, 30 × 2.1 mm with 1.8 *μ*m of particle size (SHIMADZU Corp.). The mobile phase was 2% CH_3_OH, 4.8 g/L (NH_4_)_2_CO_3_, and 98% H_2_O. The injection volume was 10 *μ*L, and the analytes of interest were isocratically separated at a flow rate of 0.4 mL/min. The quantitative analysis was conducted on the tandem mass spectrometer (MS/MS) under the following conditions: gas temperature = 200°C, sheet gas flow = 11 L/min, gas flow = 10 L/min, sheath gas heater = 350°C, capillary voltage = 3500 V, charging voltage = 50 V, and nebulizer pressure = 40 psi. All measurements were performed in duplicate using positive electrospray ionization mode (+ESI) with a sensitivity of 0.01 *μ*g/L.

### 2.7. Statistical Analysis

Data were presented as means ± standard deviation (SD). GraphPad Prism 5.01 Computer Software (GraphPad Software Inc.) was used for carrying out the statistical tests. To test the differences in seminal plasma homocysteine levels between normozoospermic males and men with asthenozoospermia, Student′s t test was performed. Differences in age between both groups were assessed using Mann–Whitney *U* test. To determine the correlations between seminal plasma homocysteine concentration, progressive and total motility, sperm concentration, semen volume, men′s age, and seminal plasma vitamin B_6_ concentration in both tested groups, Pearson′s correlation analysis was conducted. At a *p* value of < 0.05, the results were accepted as statistically significant.

## 3. Results

**Figure 1:**
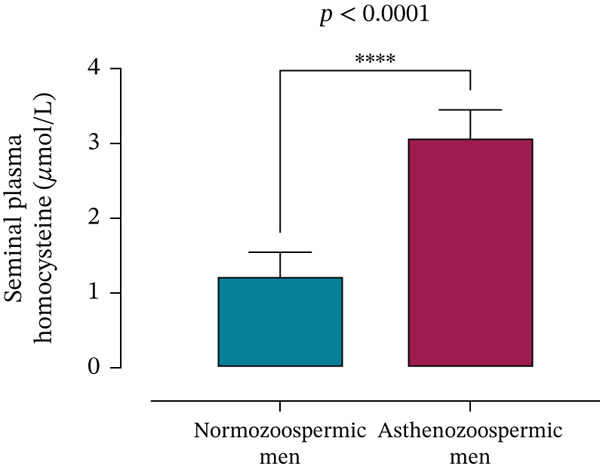
Levels of seminal plasma homocysteine in normozoospermic men (*n* = 64) compared with asthenozoospermic men (*n* = 108). Data represented as means ± standard deviation.

Figure 1 shows the homocysteine levels in seminal plasma in the normozoospermic group (1.23 ± 0.320 * μ*mol/L) compared with the asthenozoospermic group (3.08 ± 0.320 * μ*mol/L). As demonstrated in this figure, there was a highly significant difference between the mean values of the two tested groups (*p* < 0.0001).

**Figure 2:**
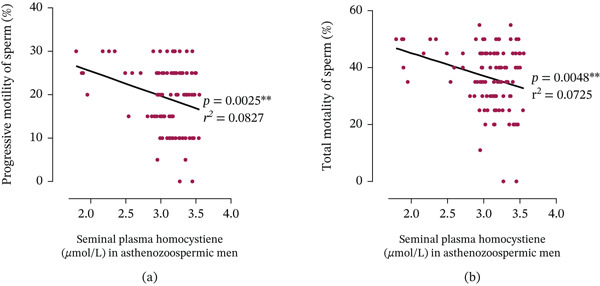
Correlation between seminal plasma homocysteine levels and (a) sperm progressive motility and (b) total motility in asthenozoospermic men.

Figure 2a,b demonstrates the correlation between seminal plasma homocysteine levels and sperm PR and total motility in asthenozoospermic men. As shown in the figure, progressive and total motility were inversely correlated with the seminal plasma levels of homocysteine (*p* = 0.0025, *r* ^2^ = 0.0827, *p* = 0.0048, *r* ^2^ = 0.0725, respectively).

**Figure 3:**
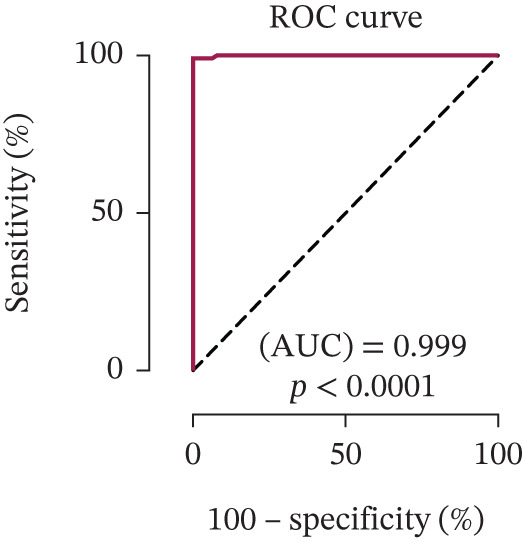
Receiver operating characteristic (ROC) curve analysis evaluating the diagnostic performance of seminal plasma homocysteine in distinguishing asthenozoospermic men from normozoospermic men.

Figure 3 presents the ROC curve analysis of seminal plasma homocysteine for distinguishing the asthenozoospermic men from the normozoospermic men. The AUC was 0.999 (*p* < 0.0001), indicating strong diagnostic performance. The cutoff value was > 1.87 *μ*mol/L, with a specificity of 100.0% (95% CI: 94.3%–100%) and a sensitivity of 99.1% (95% CI: 94.9%–99.9%).

The summary of Pearson′s correlation analysis of homocysteine levels in seminal plasma versus studied variables (semen parameters, men′s age, and seminal plasma vitamin B_6_) in both tested groups is shown in Table 1. The age difference between the studied groups is presented in Figure S1. Full sets of correlation scatter plots for each group, including nonsignificant associations, are presented in Figures S2 and S3.

**Table 1:** Summary of Pearson′s correlation analysis of homocysteine in seminal plasma versus semen parameters (progressive motility, total motility, sperm concentration, and semen volume), men′s age, and seminal plasma vitamin B_6_ in normozoospermic men and men with asthenozoospermia.

Parameter	Normozoospermic men (*n* = 64)				Men with asthenozoospermia (*n* = 108)			
	*p*	*F*	*r* ^2^	Correlation	*p*	*F*	*r* ^2^	Correlation
Men age (19–47 years)	0.00652	0.407	0.526	NS	0.157	2.028	0.0188	NS
Semen volume (mL)	0.231	1.46	0.0230	NS	0.184	1.79	0.0166	NS
Sperm concentration (10^6^/mL)	0.966	0.00187	3.01e − 005	NS	0.743	0.108	0.00102	NS
Progressive motility of sperm (%)	0.675	0.178	0.00286	NS	0.0025	9.54	0.0827	Sig.
Total motility of sperm (%)	0.178	1.86	0.0291	NS	0.0048	8.29	0.0725	Sig.
Seminal plasma vitamin B_6_ concentration (*μ*g/L)	0.749	0.103	0.00166	NS	0.924	0.00904	8.53e − 005	NS

## 4. Discussion

Homocysteine metabolism plays a significant role in various physiological and pathological functions in the male reproductive system [10, 35]. As a result, several studies have specifically focused on investigating the potential impact of homocysteine on sperm quality, including motility. Despite this, the findings remain inconsistent and inconclusive, highlighting the need for further research to clarify this association. Moreover, although numerous studies have shown that homocysteine can serve as a diagnostic marker for several diseases [36], its potential use as a diagnostic marker for male infertility, particularly in relation to sperm motility, has not yet been clearly established.

In this study, we intended to specifically evaluate the seminal plasma levels of homocysteine and their diagnostic performance in men with asthenozoospermia compared with normozoospermic men using a substantially overlapping human cohort and previously collected seminal plasma samples from our earlier study [32]. Furthermore, we intended to examine the potential correlations between homocysteine levels, sperm parameters, and seminal plasma vitamin B_6_. The study findings demonstrated that seminal plasma levels of homocysteine were significantly higher in asthenozoospermic men than in the normozoospermic men. In addition, the ROC curve analysis indicated a strong diagnostic performance of homocysteine in differentiating the asthenozoospermic from the normozoospermic group. Moreover, a robust inverse correlation between seminal plasma levels of homocysteine and both progressive and total motility of spermatozoa was observed in the asthenozoospermic group. However, this correlation was not observed in the normozoospermic men, indicating a potential association between homocysteine levels and impaired sperm motility in men with asthenozoospermia.

Several studies are in agreement with our findings. For instance, a study investigating the negative effects of homocysteine on sperm parameters, oxidative stress status, and testosterone levels in male rats reported that homocysteine treatment resulted in significantly reduced sperm motility, sperm concentration, and testosterone levels compared with controls [12]. In addition, antioxidant enzymes in plasma such as catalase, peroxidase, and superoxide dismutase were markedly reduced, whereas malondialdehyde levels were increased in homocysteine‐treated animals compared with controls [12]. A subsequent study (2015) demonstrated that direct addition of homocysteine derivatives to sperm cells induces lipid peroxidation, mitochondrial ROS generation, tyrosine phosphorylation, and suppresses sperm motility (*p* < 0.001) [11]. Rezk et al. [28] further showed that treatment of sperm cells from both normal and subfertile men with homocysteine resulted in a time‐dependent reduction in sperm motility, with differential effects between the two groups. Mechanistically, this reduction was attributed to the ability of homocysteine to induce mitochondrial superoxide anions in sperm cells, as demonstrated by flow cytometry cell sorting analysis [28]. Furthermore, a case‐control study including 353 infertile Iraqi men (asthenozoospermic, oligospermic, oligozooasthenospermic, teratozoospermic, and azoospermic) investigated the relationship between the MTHFR gene mutations, homocysteine level, and male infertility [25]. Some of the results showed that all infertile men had significantly higher serum homocysteine levels than fertile males of the same age range, with oligoasthenospermia having the highest levels (*p* < 0.01) [25]. Similarly, another human study examining the relationship between serum and seminal plasma homocysteine in relation to semen parameters in 77 infertile men (mainly oligozoospermic and asthenozoospermic men) reported higher homocysteine levels in both serum and seminal plasma compared with controls [26]. The Spearman correlation analysis further demonstrated that as the serum homocysteine level increases, the sperm concentration and velocity decreases [26].

Although previous studies have demonstrated a significant association between serum and/or seminal plasma homocysteine levels and sperm motility, several reports have yielded conflicting results, which further complicate our understanding of this relationship. For example, Ebisch et al. [27] and Kralikova et al. [31] reported that subfertile men (including asthenozoospermic men) had significantly higher homocysteine levels in sperm cells (*p* < 0.001) when compared with fertile controls; however, seminal plasma homocysteine levels were similar between the groups. Additionally, tests conducted on subfertile men undergoing IVF/ICSI procedures have shown that there were no statistically significant correlations between sperm motility and homocysteine in seminal plasma and blood [29]. In another study, the authors compared seminal plasma of total homocysteine, 15‐F2t‐isoprostane, and MDA between normozoospermic and asthenozoospermic men and examined the relationships between these parameters and sperm quality [30]. Among their findings, they found that homocysteine showed no significant difference between normozoospermic and asthenozoospermic men, and there was no correlation between homocysteine levels in seminal plasma and sperm motility [30]. Given these contradictory findings, the present study is aimed at clarifying the relationship between seminal plasma homocysteine levels and sperm motility using a larger human cohort and a sensitive analytical method such as HPLC.

**Figure 4:**
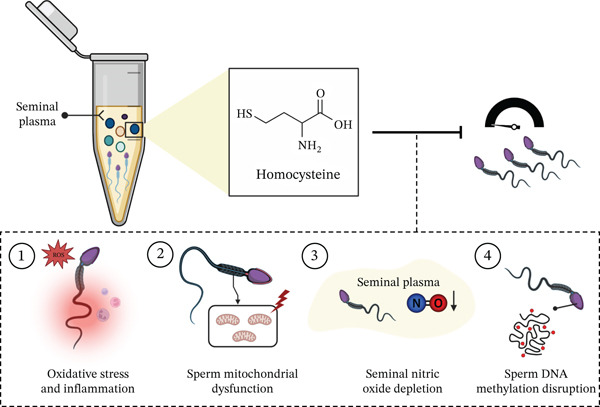
Proposed mechanisms linking homocysteine to impaired sperm motility. This figure summarizes proposed mechanisms based on previous literature and is not derived from direct mechanistic experiments performed in the present study.

The negative effects of homocysteine on sperm motility may be attributed to several underlying mechanisms, including oxidative stress and inflammation, impaired mitochondrial function, reduced seminal nitric oxide, and altered sperm DNA methylation (Figure 4). Firstly, oxidative stress is implicated in male infertility, largely through lipid peroxidation–mediated damage that compromises sperm membrane integrity and motility [37]. Homocysteine can trigger oxidative stress by generating free radicals/ROS through auto‐oxidation and nitrosylation while also disrupting antioxidant enzymes (such as SOD, CAT, and GPx) in sperm cells and seminal plasma [18, 19]. In addition, homocysteine has been linked to increased expression of proinflammatory cytokines, including MCP‐1, IL‐1, IL‐6, and IL‐8 [21, 38], as well as C‐reactive protein [22], which may disrupt immune and cytokine balance in the male reproductive tract [39]. Secondly, elevated homocysteine levels have been shown to impair sperm mitochondrial function [11]. Proposed mechanisms reported in sperm and other cellular systems include induction of mitochondrial ROS through homocysteinylation of electron transport chain proteins and disruption of ATP synthesis [28, 40, 41], as well as interference with mitochondrial membrane potential and Ca^2+^ homeostasis [42], suggesting a potential role in reducing sperm motility via mitochondrial dysfunction. Thirdly, evidence suggests that sperm motility is dependent on seminal nitric oxide concentrations [43–45]. High levels of homocysteine have been linked to reduced nitric oxide bioavailability through impairment of the nitric oxide synthase pathway [46, 47]. Given the importance of nitric oxide in maintaining normal sperm motility, it is reasonable to propose that homocysteine may impair sperm motility through nitric oxide depletion. Lastly, homocysteine regulates DNA methylation via the SAH/SAM ratio [15], and altered DNA methylation has been linked to impaired expression of genes important for sperm function and motility [48–50]. Accordingly, elevated homocysteine may disrupt DNA methylation and contribute to abnormal sperm motility. Collectively, these proposed mechanisms support a potential role for elevated homocysteine in the pathophysiology of impaired sperm motility.

The marked elevation in seminal homocysteine levels in asthenozoospermic men, together with its strong negative correlation with both progressive and total motility and its strong diagnostic performance in differentiating asthenozoospermia from normozoospermia, suggests that homocysteine may be a promising indicator of impaired sperm function. However, further research is needed to fully validate this potential biomarker. It is important to highlight that participants were recruited from a specific population, which may limit the generalizability of the findings. Another limitation is the imbalance in sample size between the asthenozoospermic and normozoospermic groups. Since sample size calculation was not performed prior to sample collection, a plausible impact on statistical power and selection bias cannot be excluded. Although ROC analysis showed strong diagnostic performance of seminal plasma homocysteine in asthenozoospermia, the high diagnostic accuracy should be interpreted with caution and warrants confirmation in independent external cohorts. In addition, the disease specificity of elevated homocysteine levels for asthenozoospermia could not be assessed, as this study did not include groups with other male reproductive disorders. As a result, larger and more balanced cohorts representing multiple male reproductive disorders should be evaluated in future studies. Lastly, the current study lacks in vitro experiments demonstrating the specific mechanisms underlying the effects of homocysteine on sperm motility. Consequently, the proposed mechanisms remain speculative and need to be confirmed by further mechanistic studies.

## 5. Conclusion

In conclusion, our study demonstrates that seminal plasma levels of homocysteine are significantly elevated in men with asthenozoospermia and are negatively correlated with sperm motility. Moreover, the ROC curve analysis indicated strong diagnostic performance of homocysteine in differentiating asthenozoospermic from normozoospermic men. These findings could pave the way for the development of a low‐cost, noninvasive biomarker to assess the risk of poor sperm motility and guide targeted treatments, ultimately improving conception outcomes for affected couples.

## Funding

This study was supported by Jordan University of Science and Technology (10.13039/501100004035; 20180156).

## Conflicts of Interest

The authors declare no conflicts of interest.

## Supporting information


**Supporting Information 1** Additional supporting information can be found online in the Supporting Information section. Figure S1: Age differences between normozoospermic and asthenozoospermic men. Figure S2: Correlations between seminal plasma homocysteine levels and semen parameters, age, and vitamin B_6_ levels in normozoospermic men. Figure S3: Correlations between seminal plasma homocysteine levels and sperm concentration, semen volume, age, and vitamin B_6_ levels in asthenozoospermic men.

## Data Availability

The data that support the findings of this study are available from the corresponding author upon reasonable request.
