# Supplementary material for: Seminal Homocysteine Levels in Men With Asthenozoospermia: Correlation With Sperm Parameters and Vitamin B6 Levels
Source: ScientificWorldJournal. 2026 Jun 9;2026:5743309. doi: 10.1155/tswj/5743309 (PMC13248781; doi:10.1155/tswj/5743309)
Supplement: Supplementary file 1 — Supporting Information 1 Additional supporting information can be found online in the Supporting Information section. Figure S1: Age differences between normozoospermic and asthenozoospermic men. Figure S2: Correlations between seminal plasma homocysteine levels and semen parameters, age, and vitamin B6 levels in normozoospermic men. Figure S3: Correlations between seminal plasma homocysteine levels and sperm concentration, semen volume, age, and vitamin B6 levels in asthenozoospermic men. [file TSWJ-2026-5743309-s001.docx]

**Seminal homocysteine levels in men with asthenozoospermia: correlation with sperm parameters and vitamin B_6_ levels**

**Supplementary Figures**

**
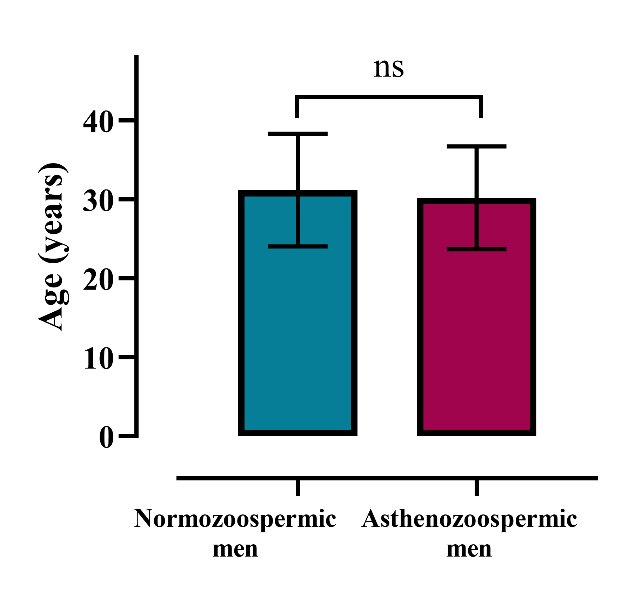
**

**Figure S1:** Age differences between normozoospermic men (n = 64) and asthenozoospermic men (n = 108). Data represented as means ± standard deviation.


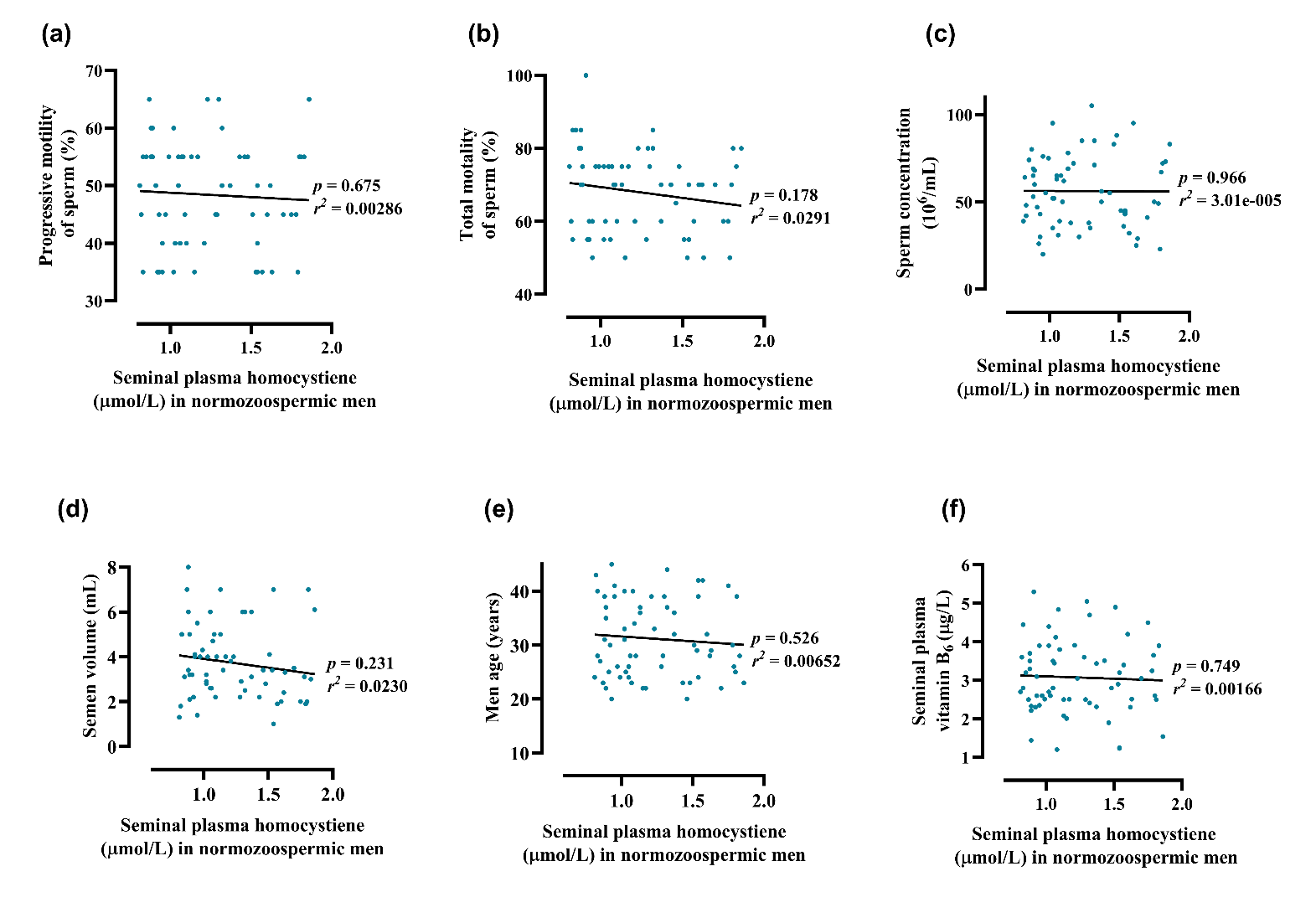


**Figure S2.** Correlations between seminal plasma homocysteine levels (µmol/L) and (a) progressive motility (%), (b) total motility (%), (c) sperm concentration (million/mL), (d) semen volume (mL), (e) age (years), and (f) seminal plasma vitamin B6 (µg/L) in normozoospermic men (n = 64).

**
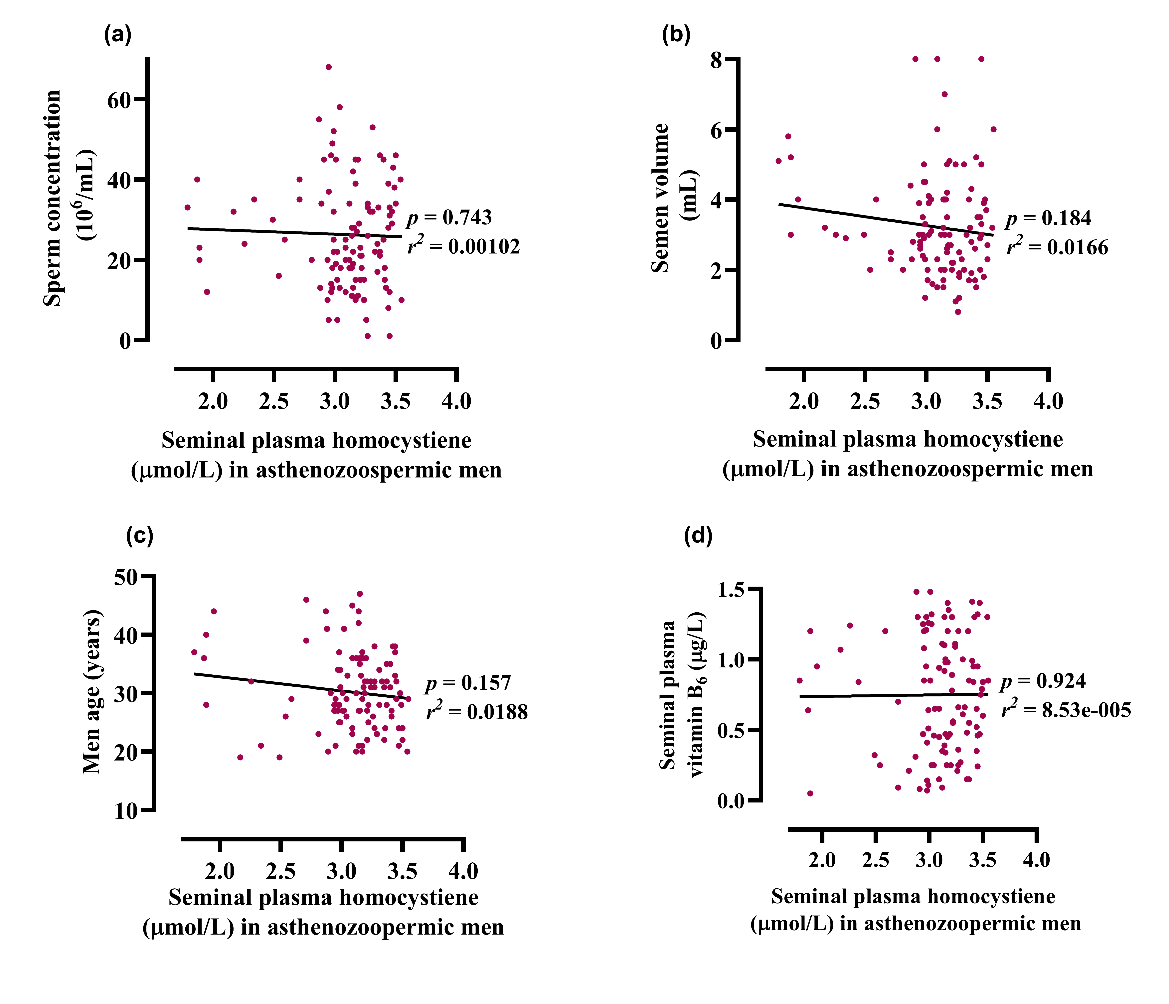
**

**Figure S3.** Correlations between seminal plasma homocysteine levels (µmol/L) and (a) sperm concentration (million/mL), (b) semen volume (mL), (c) age (years), and (d) seminal plasma vitamin B6 (µg/L) in asthenozoospermic men (n = 108).
